# Serological evidence of high exposure to *Plasmodium falciparum* gameto-cytes in dry-Season in Kenieroba, Mali

**DOI:** 10.5281/zenodo.21136984

**Published:** 2026-07-06

**Authors:** Amatigue Zeguime, Bakary Dao, Drissa Dembele, M’Bouye Doucoure, Merepen dite Agnes Guindo, Moussa Thienta, Bourama Samake, Mahamadou Hamady Assadou, Kourane Sissoko, Ismael Thera, Fanta Sogore, Adam Garango, Boucary Ouologuem, Antoine Dara, Abdoulaye K Kone, Mahamadou A Thera, Safiatou Niare-Doumbo, Laurent Dembele, Doritchamou Justin, Charles Arama, Boubacar Traore, Patrick Duffy, Abdoulaye Djimde, Bourema Kouriba, Issaka Sagara

**Affiliations:** 1 Parasites & Microbes Research & Training Center (PMRTC), International Center for Excellence in Research (ICER), University of Sciences, Techniques and Technologies of Bamako (USTTB), Bamako, Mali.; 2 Laboratory of Malaria Immunology and Vaccinology, National Institute of Allergy and Infectious Diseases (NIAID), National Institutes of Health (NIH), Bethesda, Maryland, USA.

## Abstract

**Background:**

Malaria transmission in the Sahel persists during the dry season despite low microscopic parasite prevalence, maintaining reservoirs that hinder elimination efforts. Understanding serological markers of *Plasmodium falciparum* gametocyte exposure may help identify hidden transmission reservoirs during low‑transmission periods.

**Materials and Methods.:**

We conducted a cross‑sectional survey in Kéniéroba, Mali, at three time points representing key seasonal periods: December 2024 (end of rainy season), May 2025 (dry season), and August 2025 (rainy season). A total of 356 participants were enrolled across three age groups (5-8, 9-17, and ≥18 yrs). Parasitaemia and gametocytaemia were assessed by microscopy. IgG and IgM responses to clinically isolated late-stage gametocytes were quantified by ELISA, and seropositivity thresholds defined using malaria‑naïve controls.

**Results:**

Microscopic *P. falciparum* infection prevalence remained stable across surveys (~13%), but gametocyte carriage declined from 5.7% in December to ~2% in May and August, whereas gametocyte density was higher in the latter months. IgG/IgM seropositivity exceeded 95% throughout, with IgM peaking in May, consistent with recent antigenic stimulation. Age‑stratified analyses revealed weak but statistically significant associations between antibody levels and gametocyte carriage restricted to children aged 5-8 yrs, while no associations were observed in older children or adults.

**Conclusions:**

In conclusion, serological analyses indicate widespread and seasonally variable exposure to *P. falciparum* in Kéniéroba, with consistently high IgG and IgM responses to gametocytes reflecting intense background malaria exposure and cross‑reactive immunity from asexual stages. Seasonal and age‑stratified patterns identify school‑aged children as an important reservoir sustaining transmission. Although overall correlations with gametocyte carriage were weak, age‑specific associations suggest an age‑dependent relationship between antibody responses and carriage.

## Introduction

Achieving malaria elimination requires interrupting transmission and progressively reducing parasite prevalence in targeted areas until local extinction is achieved [1,2]. The Global Malaria Eradication Programme (GMEP) successfully eliminated malaria from several regions worldwide; however, it was discontinued due to insufficient political commitment, operational challenges, and the emergence of insecticide and drug resistance [3]. Renewed global efforts, led by the World Health Organization (WHO) in collaboration with the Bill & Melinda Gates Foundation, have since prioritised malaria eradication with a strong emphasis on transmission-blocking strategies [1]. Current interventions rely primarily on insecticide-treated nets (ITNs) and artemisinin-based combination therapies, including drugs targeting sexual-stage parasites [4] and recently two malaria vaccines approved by WHO to use in children. Nevertheless, growing concerns over artemisinin resistance underscore the urgent need for innovative, sustainable, and cost-effective tools to complement existing measures [5–8].

In the Sahel, malaria transmission is highly seasonal, peaking during the rainy season and declining sharply in the dry season [9,10]. Evidence indicates that *P. falciparum* persists in human hosts during the dry season, forming a silent reservoir that seeds transmission when conditions become favourable [10–13]. Mali remains among the 11 countries with the highest malaria burden, reporting an estimated 8.2 million cases (3.1% of global cases) and 14,200 deaths (2.4% of global malaria deaths) in 2023 [14].

The sexual stage of *P. falciparum*, the gametocyte, is non-pathogenic yet indispensable for transmission. Gametocytes arise when a small fraction of asexual parasites (<10%) differentiate into sexual forms, progressing through five developmental stages (I-V) over 9-12 days. While immature stages are sequestered in bone marrow and spleen, only mature stage V gametocytes circulate in peripheral blood and are transmissible to mosquitoes. These mature forms represent less than 5% of total parasite biomass [15,16]. Seasonal malaria chemoprevention (SMC) has significantly reduced the incidence among children under five; however, transmission increasingly persists in older age groups, highlighting the need for interventions targeting asymptomatic carriers beyond the current scope of SMC [17].

Recent advances in molecular and transcriptomic profiling have improved understanding of parasite survival during the dry season. These studies indicate that *P. falciparum* adapts to the host environment by reducing virulence, modulating antigen expression, and prolonging circulation time, thereby facilitating persistent asymptomatic infections across seasonal gaps [18,19]. A recent study revealed that asymptomatic infections, not clinical malaria, are implicated to building and maintaining sexual ‑s tage immunity, yet because sexual‑stage antibodies are short‑lived, this immunity requires frequent boosting through repeated low‑level parasite exposure [20]. Studies clearly show that asymptomatic low‑density *P. falciparum* infections commonly persist throughout the dry season, even as clinical malaria cases nearly disappear [19,21,22]. Yet, data from dry-season infections remain scarce compared to rainy-season studies, and patterns vary across ecological zones, reinforcing the need for context-specific strategies [23].

Serology offers a complementary window into the dynamics of malaria transmission, capturing cumulative and recent exposure even when parasitaemia is low or undetectable by microscopy [11]. Antibodies against gametocyte antigens, including Pfs48/45 and Pfs230, have been detected at varying levels across endemic populations and may serve as biomarkers of sexual‑stage exposure [24]. However, repeated asexual‑stage *P. falciparum* infections were associated with increased serological responses to the gametocyte antigens Pfs48/45 and Pfs230, indicating that asexual exposure contributes to the acquisition of gametocyte‑specific immunity [25]. Comprehensive analyses of gametocyte‑specific proteins and transcripts have demonstrated that several antigens are uniquely expressed during gametocytogenesis and elicit antibody responses exclusively in individuals exposed to gametocytes, providing direct evidence that these antigens may serve as robust serological markers of gametocyte exposure [26,27].

In this context, the village of Kéniéroba in southern Mali provides an ideal setting to investigate dry‑season transmission dynamics. The area experiences intense seasonal transmission, followed by a prolonged dry season during which low‑level infections are thought to persist. Yet, sero‑parasitological studies across this seasonal gradient remain scarce. In this study, we aimed to develop an ELISA-based assay to identify serological signatures indicative of current or past gametocyte carriage. Using whole clinically isolated gametocytes as antigen, we assessed IgG and IgM responses in children and adults and examined their associations with gametocyte carriage detected by microscopy. By integrating parasitological and serological data, this study provides new insights into age‑structured reservoirs, seasonal exposure patterns, and the potential role of serology in detecting residual transmission during the dry season in Mali.

## Materials and Methods

This study was conducted in Kéniéroba, a rural village located approximately 70 km southwest of Bamako in the district of Kati, region of Koulikoro, Mali. The area lies within the Sudano-Guinean ecological zone, characterised by a highly seasonal malaria transmission pattern. Rainfall occurs mainly between June and November (with over 1,000 mm of annual rainfall), followed by a prolonged dry season until to May [28]. The ecological setting of the study village is highly favourable for malaria transmission. Located on an extensive alluvial plain that stretches toward the Niger River and is characterised by seasonal water accumulation. During the rainy season, numerous temporary water bodies form across the plain, which is predominantly used for rice cultivation. These aquatic habitats, combined with natural soil fissures, constitute optimal breeding sites for *Anopheles* larvae. Agricultural activities, including indoor farming during the rainy season, and the proliferation of wild grasses within village perimeters further enhance vector density and aggressiveness. The entomological fauna is dominated by invertebrates and arthropods, with *Anopheles gambiae s.l.* and *An. funestus* being the most prevalent species. These vectors play a major role in the transmission of *P. falciparum* malaria and Bancroftian filariasis, particularly during the peak rainy season when their abundance is highest. Malaria transmission peaks during the rainy season and declines sharply thereafter, although *P. falciparum* infections are supposed to persist at low densities during the dry season [29]. The population is predominantly engaged in subsistence farming, and access to health services is limited to a community health centre. Previous studies in this region have documented high malaria prevalence during the rainy season and residual carriage, moderate mosquito biting and infectivity rate during the dry season [30], making it an ideal setting to investigate sero-parasitological signatures of transmission persistence.

### Study design and population

A cross-sectional survey was carried out in December 2024, May 2025 and August 2025, corresponding to the end of rainy season (where gametocytes are more prevalent), the dry season and the rainy season, respectively. A total of 356 unique participants were enrolled. Prior to each survey, field guides informed all households in the village. Individuals from predefined age groups (5-8 yrs, 917 yrs, and adults) voluntarily presented to the survey site with their parent or guardian when applicable. Among those present, participants were selected using simple random sampling until the required sample size for each age group was reached. Demographic and clinical data, including age, sex, fever history, and recent antimalarial use, were collected using a structured questionnaire during the clinical and laboratory visits.

### Ethics statement

The protocol was approved by the Institutional Ethics Committee of the University of Sciences, Techniques and Technologies of Bamako (USTTB) (reference numbers Nº2024/200/CE/USTTB). Community permission was obtained, then all the participants, their parents and/or guardians (in the case of those under 18 yrs) provided written informed consent, and assent was obtained from older children (aged 12-17 yrs).

### Sample collection

Venous blood samples were collected in EDTA and Serum Separator Tube (SST) tubes for parasitological and serological analyses, respectively. Thick and thin blood smears were prepared immediately, air-dried, and stained with 10% Giemsa for microscopic examination. Aliquots of sera were separated by centrifugation and stored at -20°C for subsequent serological assays.

### Parasitological assessment

Assessment of parasite presence and density was performed in participants’ peripheral venous blood samples by microscopy using institutional standard operating procedures (SOP), based on WHO standards. Thick smears were examined under oil immersion (x1000 magnification) by certified microscopists. Parasitaemia and gametocytaemia were calculated by counting asexual and sexual parasites against 200 leukocytes and expressed as parasites per microlitre, assuming a standard leukocyte count of 8,000/μL.

### Serological analysis

To capture natural exposure signatures to gametocytes and schizonts, based on our previous serological assay experiences [31–33] at PMRTC, an accredited clinical laboratory since 2010, we optimised two ELISAs using whole clinically isolated gametocytes and laboratory strain 3D7 schizonts as antigens to detect and semi-quantify the IgG and IgM antibody responses in the study population. Clinical isolates of *P. falciparum* gametocytes were purified using magnetic-activated cell sorting (MACS). The whole blood from *P. falciparum* gametocytes carriers was diluted in cold suspended animation buffer or 1×PBS, loaded onto the column, and extensively washed to remove uninfected erythrocytes and leukocytes. Retained late-stage parasites were eluted in two steps, centrifuged, and resuspended in minimal buffer to maximise concentration for downstream assays. Microscopic examination of the MACS elution was performed to quantify gametocytes per microlitre and to check for the presence of any asexual parasites. No asexual *P. falciparum* parasites were detected in any of the eluted samples. Schizont-stage extracts were generated by treating *P. falciparum* infected erythrocytes with 0.03% saponin to lyse host-cell membranes while preserving intact parasites. The parasite pellet was then repeatedly washed in 1×PBS with protease inhibitors, resuspended, aliquoted, and stored at -80°C until use. To determine the optimal dilution of the antigens and the samples, we coated a 96‑well microplate using a serial dilution layout, testing the antigens across 12 points and the positive serum across 8 points. The positive serum consisted of a pool of sera from several gametocyte carriers. Using this approach, we selected 6 μL of clinical gametocyte isolates and 25 μL of the 3D7 schizont lysate for ELISA. Samples were diluted 1:50 for clinical gametocyte extracts and 1:400 for the 3D7 schizont lysate. Briefly, for study sample testing, 100 μL of clinically isolated gametocytes diluted in carbonate-bicarbonate buffer were coated onto 96-well Immulon® 4 HBX plates and incubated overnight at 4 °C. Plates were washed manually four times with 300 μL per well of wash buffer (1×PBS supplemented with 0.1% Tween-20 and 0.9% NaCl). Wells were then blocked with 300 μL of blocking buffer (3% skimmed milk in 1×PBS containing 0.1% Tween-20 and 0.9% NaCl) for 90 min at room temperature (RT) with gentle agitation, followed by washing as described above. Subsequently, 100 μL of serum samples (diluted in diluent buffer: 1% skimmed milk in 1×PBS containing 0.1% Tween-20 and 0.9% NaCl) were added in duplicate and incubated for 90 min at RT with gentle shaking. Malaria-naïve serum and a pooled serum from gametocyte-positive individuals were included on each plate as negative and positive controls, respectively. The positive control consisted of a pooled serum sample prepared from gametocyte carrier participants. Sera with the highest IgM reactivity (OD > 2.0) were selected and pooled to generate a strong, stable positive control. Across all assay plates (n = 21 readings), the positive control showed a median OD of 3.24 (IQR: 2.87-3.30) and a coefficient of variation of 12.2%, demonstrating high inter‑plate consistency. Negative controls consisted of individual sera from malaria‑naïve participants, and these samples displayed a median OD of 0.46 (IQR: 0.39-0.52) with a CV of 20.9%. Goat anti-human IgG or IgM (H+L) cross-adsorbed secondary antibodies conjugated to horseradish peroxidase (HRP; Thermo Fisher) were diluted 1:4000 in diluent buffer and incubated for 1 h at RT, followed by washing as previously described. Colour development was achieved by adding 100 μL of 1-Step Ultra TMB substrate (Thermo Fisher) and incubating for 10 min before stopping the reaction with 1 N sulfuric acid. Absorbance was measured at 450 nm and 650 nm using a SpectraMax ABS microplate reader (Molecular Devices). Background signal was corrected by subtracting optical density (OD) at 650 nm from OD at 450 nm. Seropositivity thresholds were defined using mean OD + 3 standard deviation (SD) of malaria-naïve controls.

### Data analysis

Electronic data capture was used and data were extracted in csv format. All statistical analyses were performed using RStudio version 4.3.1. Descriptive statistics summarised parasitological data and antibody responses. Continuous variables such as antibody optical density values were summarised as medians with interquartile ranges (IQR) and compared between groups using non-parametric tests (Mann-Whitney U test for two-group comparisons and Kruskal-Wallis test for multiple groups). Seasonal variations and age-stratified differences in IgG and IgM responses were assessed using these tests, followed by Dunn’s post hoc correction for multiple comparisons where appropriate. Age‑stratified associations between antibody levels and gametocyte carriage were assessed using Spearman’s rank correlation, treating antibody responses as continuous variables and gametocyte carriage as a binary outcome (positive vs. negative), with 95% confidence intervals obtained by bootstrap resampling. The IgG/IgM ratio was calculated to explore patterns indicative of recent or cumulative exposure. Categorical variables were compared using Chi-square or Fisher’s exact tests as appropriate. Significance was set at p < 0.05 for all analyses. Confidence intervals (95% CI) are reported where relevant.

## Results

### Seasonal Variation in Malaria Prevalence and Gametocyte Carriage

Malaria infection prevalence remained stable across the three survey periods (December 2024: 13% [40/299], May 2025: 13% [30/233], August 2025: 9.8% [(20/204]; p = 0.5). In contrast, gametocyte carriage showed a significant decline from December (5.7% [17/299] to May (2.1% [5/233] and August (2.0% [4/204]; p = 0.032).

Parasitaemia levels varied significantly across the survey months (p = 0.023). Median parasite densities were 360 parasites/μL (IQR 200-5,160; n = 40) in December 2024, 160 parasites/μL (IQR 80-720; n = 30) in May 2025, and 480 parasites/μL (IQR 220-2,080; n = 20) in August 2025.

Gametocyte density did not differ significantly across survey periods (p = 0.3). Median densities were 120 gametocytes/μL (IQR 80-240; n = 17) in December 2024, 120 gametocytes/μL (IQR 120-640; n = 5) in May 2025, and 4,240 gametocytes/μL (IQR 540-8,300; n = 4) in August 2025. Although August had a very small number of quantifiable carriers, these individuals exhibited markedly higher densities compared with the other survey periods (Table 1).

**Table 1.** Summary of malaria infections and parasitaemia by season.

	Dec-2024 n=299^*^	May-2025 n=233^*^	Aug-2025 n=204^*^	p-value^**^
Malaria infection				0.5
Negative	259 (87.0%)	203 (87.0%)	184 (90.2%)	
Positive	40 (13.0%)	30 (13.0%)	20 (9.8%)	
Gametocyte carriage				0.032
Negative	282 (94.3%)	228 (97.9%)	200 (98.0%)	
Positive	17 (5.7%)	5 (2.1%)	4 (2.0%)	
Parasitaemia/µl	360 [200-5160]	160 [80-720]	480 [220-2080]	0.023
Gametocytaemia/µl	120 [80-240]	120 [120-640]	4240 [540-8300]	0.3

^*^: n (%); Median [Q1-Q3].^ **^ : Pearson's Chi-squared test; Kruskal-Wallis rank sum test.

### Age-stratified patterns

*P. falciparum* malaria infection prevalence varied significantly across age groups (p < 0.001). Infection was highest among children aged 9-17 yrs (17% [44/259]) and 5-8 yrs (15% [33/227]), while adults (>18 yrs) exhibited a markedly lower prevalence (5.2% [13/250]). Gametocyte carriage also differed significantly by age (p < 0.001), with the highest prevalence in children aged 5-8 yrs (7.5% [17/227]), compared with (2.3% [6/259]) in the 9-17-yrs group and (1.2% [3/250]) in adults.

Parasitaemia levels were not significantly different across age categories (p = 0.5), though younger children (5-8 yrs) showed the highest values 560 parasites/μl (IQR 120-3,160; n= 33). Gametocyte density similarly did not differ statistically (p = 0.2), but older children had the highest absolute densities 540 gametocytes/μl (IQR 120-9,160; n= 44), suggesting that school-aged children may represent a key reservoir for transmission due to their combined high infection prevalence and elevated gametocyte burden (Table 2).

**Table 2.** Summary of malaria infections and parasitaemia by age cohort.

	5-8 years n=227*	9-17 years n=259*	≥18 years n=250^*^	p-value^**^
Malaria Infection				<0.001
Negative	194 (85.0%)	215 (83.0%)	237 (94.8%)	
Positive	33 (15.0%)	44 (17.0%)	13 (5.2%)	
Gametocyte Carriage				<0.001
Negative	210 (92.5%)	253 (97.7%)	247 (98.8%)	
Positive	17 (7.5%)	6 (2.3%)	3 (1.2%)	
Parasitaemia/µl	560 [120-3160]	280 [100-720]	360 [200-640]	0.5
Gametocytaemia/µl	120 [120-520]	540 [120-9160]	40 [40-240]	0.2

^*^: n (%); Median [Q1-Q3].^ **^ : Pearson's Chi-squared test; Kruskal-Wallis rank sum test.

### Immunogenicity to clinically isolated gametocytes

IgG and IgM responses were consistently high across visits (≥95% positivity), with no significant differences for IgG (p = 0.2) or IgM (p = 0.085).

However, the IgG/IgM ratio varied significantly across surveys (p = 0.022), and low ratios were highly prevalent in all seasons: December (84% [251/299]), May (82% [190/233]) and August (91% [185/204]) (Table 3a-c).

Given evidence of shared antigens between asexual and sexual stages, we sought to determine whether the previously observed high seropositivity is partially due to general malaria exposure rather than sexual‑stage‑specific immune activation. To address this, we evaluated IgM reactivity against an asexual schizont extract. IgM anti‑schizont positivity was consistently high across all visits (92-100%) and showed significant seasonal variation (p < 0.001), indicating intense and ongoing exposure to asexual parasites in the study population. Nevertheless, these findings do not fully exclude the presence of IgG and IgM responses directed against gametocyte‑specific antigens (Table 3d).

**Table 3.** IgG (a) and IgM (b) Anti clinical isolated gametocyte seroprevalence per visit; ratio of IgG/IgM anti CI-gametocyte confirming ongoing or recent gametocyte carriage (c); IgM Anti Schizont extract sero-

	Dec-2024 n=299^*^	May-2025 n=233^*^	Aug-2025 n=204^*^	P- value^**^
(a) IgG Anti-CI-Gametocyte				0.2
Negative	12 (4.0%)	5 (2.1%)	3 (1.5%)	
Positive	287 (96.0%)	2287.9%)	201 (98.5%)	
(b) IgM Anti-CI-Gametocyte				0.085
Negative	16 (5.4%)	4 (1.7%)	7 (3.4%)	
Positive	283 (94.6%)	2298.3%)	197 (96.6%)	
(c) Ratio IgG/IgM				0.022
High	48 (16.0%)	43 (18.0%)	19 (9.0%)	
Low	251 (84.0%)	190 (82.0%)	185 (91.0%)	
(d) IgM Anti-Schizont extract				<0.001
Negative	22 (7.4%)	1 (0.4%)	16 (7.8%)	
Positive	277 (92.6%)	232 (100%)	187 (92.2%)	

^*^: n (%). ^**^ : Pearson's Chi-squared test.

### Seasonal Dynamics of Antibody Levels

**Figure 1:**
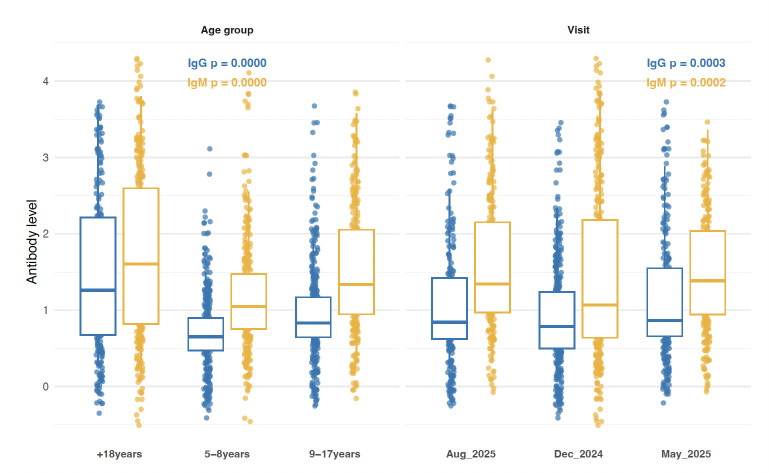
Dynamics of IgG (blue), IgM (orange) anti clinical gametocytes levels by season and age groups (Kruskal-Wallis p-values shown).

Relative quantitative antibody levels differed significantly across visits for both IgM (p = 0.0002) and IgG (p = 0.0003). IgM levels were consistently higher than IgG, peaking in May, reflecting recent antigenic stimulation. IgG showed less fluctuation, consistent with long-lived immunity (Figure 1).

### Age-dependent antibody responses

Antibody levels increased with age for both IgG and IgM (p < 0.0001), with the lowest medians in 58 years and highest in adults (≥18 years) indicating cumulative exposure and immune maturation (Figure 1).

**Association between antibody level and gametocyte carriage**

**Figure 2:**
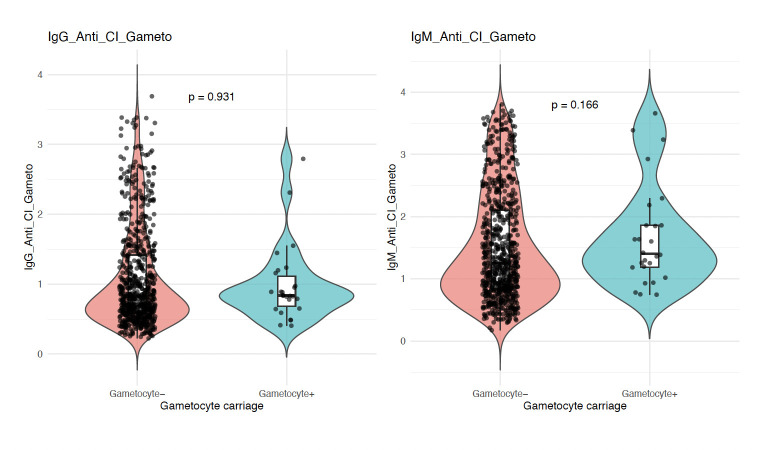
Association between IgG, IgM anti CI-Gametocytes and Gametocyte carriage.

Antibody levels did not differ significantly between gametocyte-positive and gametocyte-negative individuals for IgG (p = 0.931) or IgM (p = 0.166). Persistent high IgG and IgM levels in gametocyte-negative individuals suggest long-lived immunity and repeated exposures. Slightly higher IgM in gametocyte-positive individuals may indicate recent stimulation, but variability and overlap limit its predictive value in the context of Kéniéroba village. These findings indicate that absolute relative antibody levels against gametocytes are poor markers of active infection, and longitudinal measures may better capture recent exposure (Figure 2).

### Correlation between antibodies and gametocyte carriage

**Figure 3:**
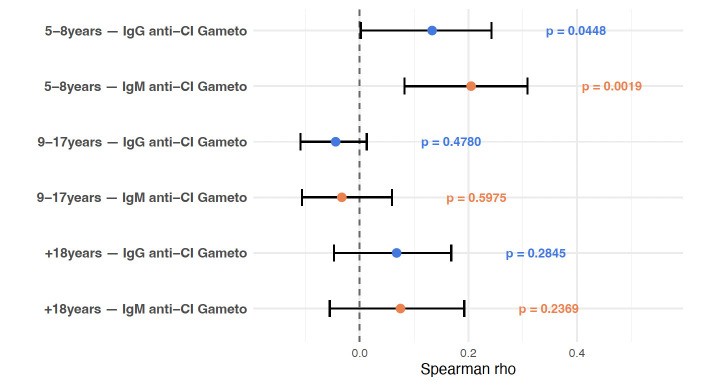
Age‑stratified associations between CI-Gametocytes antibody responses and gametocyte carriage. For details, see text.

Overall, antibody levels were not associated with microscopy‑detected gametocyte carriage. However, age‑stratified analyses revealed a limited pattern: in children aged 5-8 years, IgM showed a weak but statistically significant correlation with gametocyte density (r = 0.21, p = 0.0019), and IgG demonstrated an even weaker association (r = 0.13, p = 0.0448). In older children and adults, correlations were negligible and non‑significant. These findings indicate that while antibody responses may reflect recent exposure in the youngest age group, they primarily represent cumulative exposure rather than current gametocyte carriage in the broader population. (Figure 3). Spearman’s rank correlation coefficients were used to evaluate age specific associations between antibody responses to clinically isolated *P. falciparum* gametocyte and gametocyte carriage. Antibody responses were analysed as continuous variables while gametocyte carriage was encoded as a binary variable (1 = gametocyte positive; 0 = gametocyte negative), such that the correlation measures whether higher antibody levels tend to be observed in individuals carrying gametocytes compared with non carriers within each age group (5-8 yrs, 9-17 yrs, and ≥18 yrs).

Points represent the estimated Spearman correlation coefficients for each antibody-age group combination, with horizontal bars showing 95% confidence intervals derived from bootstrap resampling. Corresponding p values are displayed alongside each estimate. Positive values indicate that higher antibody levels are associated with a greater likelihood of gametocyte carriage, whereas values near zero indicate little or no monotonic association.

Associations were weak and restricted to the youngest age group (5-8 yrs), where IgM and to a lesser extent IgG showed modest but statistically significant correlations with gametocyte carriage. No significant associations were detected in older children or adults. Because gametocytaemia was highly zero inflated in this cohort, treating gametocyte status as either a binary or continuous variable yielded comparable rank based associations; thus, the correlations primarily reflect differences between gametocyte positive and negative individuals rather than variation in gametocyte density among carriers.

## Discussion

### Seasonality and dry season reservoirs

In Kéniéroba, microscopy‑defined *P. falciparum* prevalence remained stable across surveys, but gametocyte carriage declined markedly from December to May and August. Notably, May corresponds to the middle of the dry season, when transmission is expected to be minimal, yet gametocyte carriage persisted and densities remained measurable, indicating ongoing maturation of sexual stages and chronic low‑level infections that maintain the reservoir. Although differences in gametocyte density across surveys were not statistically significant, August showed clearly higher absolute densities alongside the highest parasitaemia, suggesting renewed transmission with the onset of rains. Together, these patterns point to a seasonal shift in the composition of the infectious reservoir: during the dry season, a smaller number of chronically infected individuals with low‑to‑moderate densities sustain transmission potential, which then expands as rains return. This aligns with evidence of dry‑season reservoirs, submicroscopic persistence, and parasite adaptations that bridge transmission seasons in Sahelian settings [10–12,14].

### Age specific reservoirs

Age-stratified analyses point to school-aged children as the epidemiological fulcrum: infection prevalence was highest in 9-17 yrs cohort (followed by the 5-8 yrs cohort), adults had substantially lower prevalence, gametocyte carriage was most frequent in the youngest (5-8 yrs), and the highest gametocyte densities were observed among older children (9-17 yrs). Median parasitaemia did not differ significantly across age groups, but absolute values were greatest in younger children. This configuration (more frequent carriage in the youngest and higher densities in older children) is compatible with shifting age-specific immunity and control pressure (e.g., SMC), and mirrors findings that school-aged children can disproportionately fuel transmission [14,17,34].

### High seropositivity without evidence of association with gametocyte carriage

Using clinically isolated gametocytes, IgG and IgM seropositivity was ≥95% across all surveys, indicating broad humoral recognition of sexual‑stage antigens under natural exposure [15,16,24]. Seasonal fluctuations were observed [11,35] in both antibody classes, with IgM consistently exceeding IgG and peaking in May. Although IgG and IgM levels varied seasonally and low IgG/IgM ratios were common, we did not observe any association between antibodies level and microscopy‑detected gametocyte carriage. Moreover, because asexual and gametocyte stages share antigens [16,25,36], and because serological markers may persist for several months after exposure, the high IgG and IgM positivity observed in this study may partly reflect repeated or recent malaria exposure rather than ongoing gametocyte carriage.

To evaluate whether the observed serological patterns reflected general malaria exposure rather than gametocyte-specific immune activation, IgM responses to a 3D7 strain schizont extract was measured by ELISA. IgM‑anti‑schizont positivity was extremely high across all surveys (92-100%) and exhibited significant seasonal variation, confirming intense ongoing asexual exposure. However, the temporal patterns of schizont‑specific IgM did not fully mirror those of gametocyte‑specific IgG and IgM. This small divergence suggests that, although cross‑reactivity is substantial as expected when using whole‑gametocyte antigen preparations, the sexual‑stage antigen extract captures immunological signals that cannot be entirely explained by exposure to asexual stages. Further investigations will be required to more clearly distinguish antibodies elicited by asexual‑stage exposure from those attributable to gametocyte‑specific immunological responses.

### Antibody associations and correlation with gametocyte carriage

Cross‑sectional antibody levels did not differentiate gametocyte‑positive from gametocyte‑negative individuals, indicating that absolute titres are not reliable markers of active gametocyte carriage in this high‑exposure setting. Age‑stratified analyses revealed limited associations only in the youngest children (5-8 yrs), where IgM showed a weak but statistically significant correlation with gametocyte carriage, and IgG an even weaker association. No correlations were detected in older children or adults. These weak, age‑restricted relationships suggest that antibody responses may partially reflect recent exposure in younger individuals, while in older age groups they mainly capture cumulative exposure rather than current gametocyte carriage.

### Public health implications

Although serology cannot identify active gametocyte carriers, the seasonal shifts in antibody patterns together with parasitological data highlight population‑level trends in malaria exposure. The concentration of infection and gametocyte burden in school‑aged children reinforces their importance for targeted malaria control [1,2]

### Strengths and limitations

By integrating microscopy, gametocyte measurements, and sexual‑stage serology across seasons and age groups, this study provides a detailed view of local transmission dynamics and identifies who drives transmission and when. Using late‑stage clinical gametocytes enhances ecological relevance compared with recombinant proteins [15,16]. Key limitations include the use of microscopy, which is less sensitive for low‑density infections [12], and the examination of only 200 WBC, which may further reduce sensitivity for detecting very low‑density gametocytes. The cross sectional design limits causal inference and temporal resolution. Importantly, because whole gametocytes contain shared epitopes with asexual parasites [25,36], cross‑reactive antibodies almost certainly contributed to observed responses. Moreover, cryptic antigens within clinical gametocytes may not be fully accessible, reducing capture of antibodies to hidden epitopes. Despite MACS enrichment, residual non‑gametocyte material cannot be excluded.

### Future directions

Comparative analyses using recombinant antigens (e.g., Pfs230 and Pfs48/45) may improve stage specificity and help define serological signatures associated with gametocyte exposure [16,24]. Assessing IgG subclasses may also elucidate functional antibody responses, particularly given links to transmission‑reducing activity [33].

## Conclusions

In conclusion, serological analyses revealed widespread and seasonally variable exposure to *P. falciparum* in Kéniéroba, with consistently high IgG and IgM responses to gametocyte antigens. Uniformly elevated IgM reactivity to schizont extract indicates intense background exposure to asexual parasites, suggesting that a substantial proportion of the observed antibody responses reflects cross‑reactive immunity rather than strict gametocyte specificity. Seasonal antibody dynamics, together with the concentration of infections and carriage among school‑aged children, highlight critical populations and periods that sustain transmission. Collectively, these findings underscore the limitations of microscopy and single time‑point serological measurements for identifying active carriage.
